# The complete mitochondrial genome of the Basidiomycete fungus *Pleurotus cornucopiae* (Paulet) Rolland

**DOI:** 10.1080/23802359.2017.1422405

**Published:** 2018-01-03

**Authors:** Li-Ming Xu, Damien Daniel Hinsinger, Guo-Feng Jiang

**Affiliations:** a Biology Institute, Guangxi Academy of Sciences, Nanning, Guangxi, PR China;; b Biodiversity Genomics Team, Plant Ecophysiology & Evolution Group, Guangxi Key Laboratory of Forest Ecology and Conservation, College of Forestry, Guangxi University, Nanning, Guangxi, PR China;; c State Key Laboratory for Conservation and Utilization of Subtropical Agro-Bioresources, Guangxi University, Nanning, Guangxi, PR China

**Keywords:** Fungi, mushroom, *Pleurotus cornucopiae*, mitogenome, evolution

## Abstract

*Pleurotus cornucopiae* is a commercial edible and medicinal fungus. Herein, we determined and analyzed its complete mitochondrial genome. The mitogenome length was 72,134 bp with a GC content of 26.7%, contained 14 conserved protein coding genes, two rRNA genes (*rnl* and *rns*), ribosomal protein gene *rps3* and 24 tRNA genes. *Pleurotus cornucopiae* has a similar gene content and gene order of the mitogenome as *P. ostreatus* and *P. eryngii*. A phylogenetic analysis based on complete mitogenome in related fungi showed that *P. cornucopiae* is a member of the order Agaricales, forming a clade with *P. ostreatus* and *P. eryngii*, with *P. ostreatus* as a sister taxa. The mitochondrial genome sequence of *P. cornucopiae* appeared a promising tool for further studies of the taxonomy and evolution of Pleurotaceae and Agaricales.

The genus *Pleurotus,* belonging to the family Pleurotaceae (Agaricales, Agaricomycetes, Basidiomycota), is one of the most diverse group of cultivated mushrooms in the world. The *Pleurotus* species establish a wide range of interactions with other organisms, and are efficient bioconverters of lignocellulosic residues into human food (Tsuneda and Thorn 1995; Philippoussis 2009). The biodiversity of *Pleurotus* remains highly investigated and recently, molecular studies have increase our knowledge about intra and inter-specific heterogeneity of the genus *Pleurotus* using both ribosomal and mitochondrial DNA (Li and Yao 2004; Wang et al. 2008; Yang et al. 2016; Chaudhary and John 2017). The edible fungus *Pleurotus cornucopiae* is emerging as an important species in the genus, with isolation of medicinal metabolites from the fruiting body (Wang et al. 2012). The genetic diversity and population structure of *P. cornucopiae* is well known (Iraçabal and Labarere 1994; Zervakis et al. 1994; Shnyreva et al. 1996; Zhang et al. 2007; Zhou et al. 2009; Adebayo et al. 2016); however, no complete mitogenome is available to date for the species. Here, we report the complete mitogenome of *P. cornucopiae* (GenBank accession MG652610) to provide new genetic resources and performed a phylogenetic analysis of related taxa of this species.

The strain SWS_15 of *Pleurotus cornucopiae* is maintained in the Biology Institute, Guangxi Academy of Sciences (Nanning, Guangxi, PR China). Total genomic DNA was extracted as previously described (Xu et al. 2017). Library construction and sequencing were processed by Novogene (Beijing, China), according to the Illumina HiSeqX-ten system manufacturer instructions (Illumina, San Diego, CA). The mitogenome of *Pleurotus cornucopiae* was *de novo* assembled using ORG.Asm v0.2.05 (https://pythonhosted.org/ORG.asm/) followed by manual curation in Geneious R9 v9.1.6 (Biomatters Ltd, Auckland, New Zealand) as described previously (Hinsinger and Strijk 2016; Jiang et al. 2016; Xu et al. 2017). Genome annotation and phylogenetic analysis were performed with DOGMA (http://dogma.ccbb.utexas.edu/index.html) and Phyml 3.1 (Guindon et al. 2010), respectively. Maximum-likelihood (ML) tree was constructed including 10 available mitogenomes of Agaricales and *Heterobasidion irregulare* as an outgroup (Figure 1).

**Figure 1.**
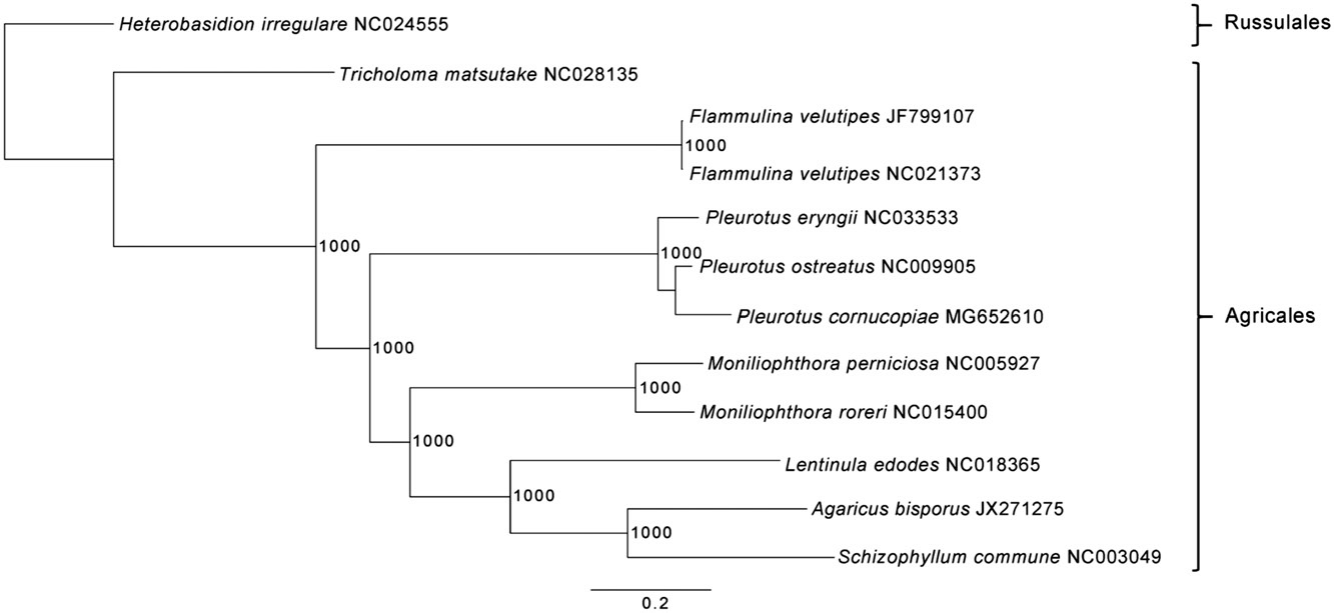
ML phylogenetic tree of the 10 available mitogenomes of Agaricales in GenBank, plus the mitogenome of *Pleurotus cornucopiae*. The tree is rooted with *Heterobasidion irregulare*. Bootstraps values (1000 replicates) are shown at the nodes. Scale in substitution per site.

The complete mitogenome of *Pleurotus cornucopiae* was 72,134 bp in length with a GC content of 26.7%. The mitogenome contained 14 conserved protein coding genes, 2 rRNA genes (*rnl* and *rns*), the ribosomal protein gene *rps3* and 24 tRNA genes. The 14 conserved protein coding genes respectively encoded the seven ubiquinone reductase subunits of NADH (*nad1*, *nad2*, *nad3*, *nad4*, *nad4L*, *nad5* and *nad6*), three cytochrome oxidase subunits (*cox1*, *cox2* and *cox3*), three ATP synthase subunits (*atp6*, *atp8* and *atp9*) and the apocytochrome b (*cob*). The 24 tRNA genes ranged in size from 71 bp to 93 bp, and covered all 20 standard amino acids. Phylogenetic analysis based on ML showed with high support that *Pleurotus cornucopiae* is a member of Agaricales and grouping with *P. ostreatus* and *P. eryngii*, and closely related to *P. ostreatus* (Figure 1). This result is consistent to the previous study based on SSU rDNA or LSU rDNA analyses (Gao et al. 2008; Chaudhary and John 2017), but differs from the result based on ITS data (Shnyreva and Shnyreva 2015; He et al. 2016). The complete mitochondrial genome of *P. cornucopiae* provides the community a useful bioresource that will help to delineate taxonomic units in *Pleurotus*, and will be of interest for further applied researches by selecting the most suitable species for biotechnological and nutritional interest.
